# ARE THERE DIFFERENCES IN LAPAROSCOPIC GASTRECTOMY MORBIDITY AND MORTALITY BETWEEN YOUNG AND OLDER?

**DOI:** 10.1590/0102-672020210002e1617

**Published:** 2021-12-17

**Authors:** Vinicius Riberio LEDUC, Fernando Augusto de Vasconcellos SANTOS, Paula Segato Vaz de OLIVEIRA, Gabrielle Stéphanie de Paula da LOMBA, Gabriela Dias de FIGUEIREDO, Joana Pereira KALIL, Alberto Julius Alves WAINSTEIN, Ana Paula DRUMMOND-LAGE

**Affiliations:** 1 Faculty of Medical Sciences of Minas Gerais, Belo Horizonte, MG, Brazil; 2 Oncad Surgical Oncology, Belo Horizonte, MG, Brazil

**Keywords:** Gastric cancer, Gastrectomy, Aged, Medical Oncology, Surgical Oncology, Câncer Gástrico, Gastrectomia, Idoso, Oncologia Clinica, Oncologia Cirúrgica

## Abstract

**Background:**

Due to the longer life expectancy and consequently an increase in the elderly population, a higher incidence of gastric cancer is expected in this population in the coming decades.

**Aim:**

To compare the results of laparoscopic GC surgical treatment between individuals aged<65 years (group I) and ≥ 65 years (group II), according to clinical, surgical, and histopathological characteristics.

**Methods:**

A observational retrospective study was performed by analyzing medical charts of patients with gastric cancer undergoing total or subtotal laparoscopic gastrectomy for curative purposes by a single oncologic surgery team.

**Results:**

Thirty-six patients were included in each group. Regarding the ASA classification, 31% of the patients in group I was ASA 1, compared to 3.1% in group II. The mean number of concomitant medications in group II was statistically superior to group I (5±4.21 x 1.42±3.08, p<0.001). Subtotal gastrectomy was the most performed procedure in both groups (69.4% and 63.9% in groups I and II, respectively) due to the high prevalence of distal tumors in both groups, 54.4% group I and 52.9% group II. According to Lauren's classification, group I presented a predominance of diffuse tumors (50%) and group II the intestinal type (61.8%). There was no difference between the two groups regarding the number of resected lymph nodes and lymph node metastases and the days of hospitalization and mortality.

**Conclusion:**

Laparoscopic gastrectomy showed to be a safe procedure, without a statistical difference in morbidity, mortality, and hospitalization time between both groups.

## RESUMO

**Racional:**

Devido à maior expectativa de vida, e consequentemente aumento da população de idosos, é esperada uma maior incidência de câncer gástrico nesta população nas próximas décadas.

**Objetivos:**

Comparar os resultados do tratamento cirúrgico por via laparoscópica do câncer gástrico entre pacientes com idade <65 anos (grupo I) e ≥65 anos (grupo II), de acordo com características clínicas, cirúrgicas e histopatológicas.

**Métodos:**

Foi realizado um estudo retrospectivo, observacional baseado na análise de prontuários médicos de pacientes com câncer gástrico, submetidos à gastrectomia total ou subtotal laparoscópica com finalidade curativa, por uma única equipe de cirurgia oncológica.

**Resultados:**

Foram avaliados 36 pacientes em cada grupo. Em relação à classificação ASA, 62,1% dos pacientes do grupo I eram ASA 1 comparado a 3.1% no grupo II. O número médio de medicações concomitantes do grupo II foi superior ao grupo I (5±4,21 x 1.42±3,08, p<0.001). A gastrectomia subtotal foi o procedimento mais realizado nos dois grupos (69,4% e 63,9% nos grupos I e II respectivamente) devido a maior prevalência de tumores distais em ambos os grupos, 54.4% grupo I e 52.9% grupo II. De acordo com a classificação de Laurén, no grupo I houve predomínio de tumores difusos (50,0%) e no grupo II do tipo intestinal (61,8%). Não houve diferença entre os dois grupos em relação a quantidade de linfonodos ressecados e de linfonodos positivos para metástases, assim como o tempo de permanência no CTI, dias de hospitalização e mortalidade.

**Conclusão:**

A gastrectomia por via laparoscópica é procedimento seguro, sem haver diferenças em morbidade, mortalidade e tempo de internação entre pacientes jovens e idosos.

## INTRODUCTION

Gastric cancer (GC) is a common and lethal type of neoplasm worldwide. It is the 3^rd^ most frequent malignant tumor among Brazilian men and the 5^th^ among Brazilian women. The overall prediction is that, by 2025, both the incidence of GC and its mortality decrease, with an increase in survival, reaching 30% in five years^25^.

The etiology is multifactorial, and among the known risk factors is the increase in age^10^. According to data from the Brazilian Institute of Geography and Statistics (IBGE), by the year 2025, Brazil should have the 6^th^ largest elderly population globally, with approximately 32 million people, which will mean almost 13% of the Brazilian population^4^.

Among the elderly, age itself is a predictor of morbidity and mortality risk, leading to an increase of 1.35 in mortality risk at 30 days after non-cardiac surgery every decade^21^.

Radical gastrectomy is the only therapeutic approach capable of curing GC, and its application should be discussed regardless of age. It is a surgical procedure in which total or subtotal gastric resection is performed, with satisfactory surgical margins, associated with D2 lymphadenectomy, the resection of the perigastric extra-perigastric lymph nodes. This is considered the standard surgical procedure for the treatment of advanced GC^1^.

Previous studies have shown that distal and total laparoscopic gastrectomy is safe among the elderly^15^ ^,^ ^26^. However, the evidence regarding GC management is scarce in this age group and the Brazilian population.

Therefore, given the higher incidence of CG in elderly populations and the progressive aging of the global population, especially in developing countries such as Brazil, it is necessary to research this medical issue more deeply.

This study aimed to investigate the short-term surgical morbimortality of laparoscopic GC gastrectomy in elderly patients compared to non-elderly patients to determine the safety, viability, and risk factors for postoperative complications associated with the surgical procedure.

## METHODS

 The Institutional Review Board approved this project following Resolution No. 466/2012 of the National Health Council under CAAE 49971615.3.0000.5134.

A cross-sectional, retrospective study was carried out by analyzing medical records and anatomopathological reports of patients with GC who underwent radical, subtotal, and total gastrectomy using a laparoscopic procedure for curative purposes in 5 years, attended by a single oncological surgery team. Patients with a diagnosis of GC of both genders were eligible. Patients with peritoneal carcinomatosis and distant metastases were excluded, which would contraindicate radical gastrectomy. Patients were stratified according to age as adults (≥18 and <65 years) and elderly (≥65 years) according to World Health Organization (WHO) criteria^29^.

The following variables were collected: 1) clinical-surgical: gender, age, smoking history, concomitant medications, surgical risk classification according to the American Society of Anesthesiology (ASA), anesthetic technique, the extent of gastrectomy (total or subtotal), length of stay in an intensive care unit (ICU), days of hospitalization, post-surgical complications, and mortality in the postoperative period; 2) histological findings: Lauren classification, tumor size, surgical margin, tumor location, number of resected lymph nodes and number of positive lymph nodes, presence of vascular, lymphatic and neural invasion, and staging. 

### Statistical analysis

Quantitative variables were presented as mean ± standard-deviation and were submitted to the Shapiro-Wilk normality test. To verify the association between two categorical variables, the chi-square tests of independence and Fisher exact and binary logistic regression model were used. The comparison of quantitative variables between the two groups was performed using the Wilcoxon Mann-Whitney test for independent samples. The analyses were developed in program R and were considered significant p<0.05.

## RESULTS

A total of 72 medical charts, 36 patients aged <65 years (group I), and 36 patients aged ≥ 65 years (group II) were evaluated. Group I had 63.9% of women. In group II, most of the sample consisted of men. Group II had a higher frequency of smokers when compared to group I (45.5% vs. 4.2%, p<0.001).

Regarding the ASA classification, 93.2% of the patients were ASA 1 and 2 in group I. Among individuals in group II, only 3.1% were ASA 1 and 59.4% ASA 2. The concomitant medications recorded in the medical charts were related to the treatment of psychiatric, cardiovascular, digestive, and endocrine comorbidities, and the use of antiparkinsonian and anti-inflammatory drugs was also reported. The mean number of concomitant medications in group II was statistically higher when compared to group I (p<0.001, Table 1).

**TABLE 1:** Clinical characteristics of patients undergoing radical gastrectomy for the treatment of gastric carcinoma (n=72)

Characteristics	Group I - <65 years (n=36)	Group II - ≥6 years (n=36)	p-value
Gender			0.018^Q^
Female	23 (63.9%)	12 (33.3%)	
Male	13 (36.1%)	24 (66.7%)	
Smoking*			<0.001^F^
No	23 (95.8%)	18 (54.5%)	
Yes	1 (4.2%)	15 (45.5%)	
ASA*			<0.001^L^
I	9 (31%)	1 (3.1%)	
II	18 (62.1%)	19 (59.4%)	
III	2 (6.9%)	12 (37.5%)	
Nº of medications in use	1.42 ± 3.08	5 ± 4.21	<0.001^W^
Psychiatric	4 (11.1%)	11 (30.6%)	0.079^F^
Antiparkinsonians		1 (2.8%)	1.000^F^
Digestive	2 (5.6%)	5 (13.9%)	0.429^F^
Endocrine	-	12 (66.7%)	<0.001^F^
Cardiovascular	7 (19.4%)	23 (63.9%)	<0.001^F^
Anti-inflammatory	-	7 (19.4%)	0.011^F^

*=variables have missings; ^Q^=chi-square test of independence, ^F^=Fisher's exact test; ^L^=binary logistic model; ^W^=Wilcoxon Mann-Whitney test for independent samples; ASA=American Society of Anesthesiologists

Among the individuals in group I, the primary histological type, according to the Laurén classification, was the diffuse type (50.0%), while in group II, the intestinal type was predominant (61.8%). When evaluating the depth of tumor involvement in the gastric wall, there was no statistically significant difference between the two groups. There was a predominance of T4 tumors in group I (37.9%) and T3 in group II (41.4%). In the majority, in both groups, the surgical specimens' margins were free of disease, representing 96.7% and 93.3% of the total cases, respectively, in groups I and II.

In the individuals of group I, the mean number of resected lymph nodes was 31.5±12.5, with 4.4±7.5 positives lymph nodes. Among the patients in group II, the mean number of resected lymph nodes was 36.4±16.2 and 7.7±14.4 positives for metastasis.

The presence of lymphatic (51.9%), vascular (56.0%), and neural (52.0%) invasion was observed in group I. Similar results were found in group II: lymphatic invasion (62.5%), vascular invasion (65.6%), and neural invasion (64.5%). There was no statistically significant difference between the two groups.

Regarding tumor staging, among group I individuals, the most common staging was IA and IIIA, representing 25.0% of the cases. Among the patients in group II, the primary tumor stages were IIB and IIIC (20.7% each, Table 2).

**TABLE 2:** Histopathological data from the surgical specimens of patients submitted to radical gastrectomy for the treatment of gastric carcinoma (n=72)

Characteristics	Group II - <65 years (n=36)	Group II - ≥65 years (n=36)	p-value
Laurén classification*			0,052^L^
Diffuse	14 (50%)	11 (32,4%)	
Intestinal	9 (32,1%)	21 (61,8%)	
Mixed	5 (17,9%)	2 (5,9%)	
Tumor*			0,403^L^
T1	7 (24,1%)	5 (17,2%)	
T2	5 (17,2%)	3 (10,3%)	
T3	6 (20,7%)	12 (41,4%)	
T4	11 (37,9%)	9 (31%)	
Margins*			1,000^F^
Involved	1 (3,3%)	2 (6,7%)	
Not involved	29 (96,7%)	28 (93,3%)	
Resected LN number*	31,5 ± 12,5	36,4 ± 16,2	0,242^W^
Positive LN number*	4,4 ± 7,5	7,7 ± 14,4	0,700^W^
Lymphatic invasion*			0,575^Q^
No	13 (48,1%)	12 (37,5%)	
Yes	14 (51,9%)	20 (62,5%)	
Vascular invasion*			0,641^Q^
No	11 (44%)	11 (34,4%)	
Yes	14 (56%)	21 (65,6%)	
Neural invasion*			0,501^Q^
No	12 (48%)	11 (35,5%)	
Yes	13 (52%)	20 (64,5%)	
Staging*			0,666^L^
IA	7 (25%)	5 (17,2%)	
IB	1 (3,6%)	2 (6,9%)	
IIA	1 (3,6%)	1 (3,4%)	
IIB	5 (17,9%)	6 (20,7%)	
IIIA	7 (25%)	4 (13,8%)	
IIIB	2 (7,1%)	4 (13,8%)	
IIIC	2 (7,1%)	6 (20,7%)	
IV	3 (10,7%)	1 (3,4%)	

*=variables have missing; ^Q^=chi-square test of independence; ^F^=Fisher's exact test; ^L^=binary logistic model; ^W=^Wilcoxon Mann- Whitney test for independent samples; value of p>0,005; LN=lymph nodes; T1=tumor invades the lamina propria or muscular mucosa (T1a) or submucosa (T1b); T2=tumor invades the muscle itself; T3=tumor invades the subserosa; T4=tumor perforates serosa (T4a) or invades adjacent structures (T4b).

Regarding the type of gastrectomy, most patients in the two groups underwent subtotal gastrectomy. There was a predominance of the tumor in the distal location (54.3% and 52.9% in groups I and II respectively), and a similar distribution concerning the proximal and medial locations. Most of the patients in group I were submitted to general anesthesia (70.6%), while in group II, most were submitted to the association of general anesthesia and regional anesthetic block (60.7%, Table 3).

Evaluating the ICU stay time, the average time was 3.58±6.86 and 4.50±5.67 days for the patients in groups I and II, respectively, with no statistical difference. Group II had a higher mean of total hospitalization days than group I (12.65±10.44 vs.9.61±7.83), with no statistical difference (p=0.608). Individuals in group II presented higher surgical morbidity compared to group I (0.75±1.00 vs. 0.56±1.08). Among the individuals in group I, the main postoperative complication was gastrointestinal (22.2%), followed by cardiorespiratory (11.1%), and with fair distribution about sepsis and renal complications (8.3%). No patient presented complications of the surgical wound in this group. In group II, the most common postoperative complications were renal (19.4%), followed by sepsis (13.9%), and cardiorespiratory and gastrointestinal complications (11.1% both). Still, in group II, the postoperative wound complications were registered in 5.6% of the patients.

The percentage of patients who evolved to death in groups I and II was 5.6% and 19.4%, respectively, without a statistical difference (Table 3).

**TABLE 3:** Surgical and postoperative data of patients undergoing radical gastrectomy for the treatment of gastric carcinoma (n=72)

Characteristics	Group I - < 65 years (n=36)	Group II - ≥ 65 years (n=36)	p-value
Anesthetic Technique*			0.065^F^
General	12 (70.6%)	11 (39.3%)	
General + Block	5 (29.4%)	17 (60.7%)	
Gastrectomy			0.84^Q^
Total	11 (30.6%)	13 (36.1%)	
Subtotal	25 (69.4%)	23 (63.9%)	
Location*			0.936^L^
Proximal	8 (22.9%)	7 (20.6%)	
Distal	19 (54.3%)	18 (52.9%)	
Medial	8 (22.9%)	9 (26.5%)	
Days of hospitalization in ICU	3.58 ± 6.86	4.50 ± 5.67	0.094^W^
Days of hospitalization*	9.61 ± 7.83	12.65 ± 10.44	0.609^W^
Number of complications	0.56 ± 1.08	0.75 ± 1.00	0.160^W^
Cardiorespiratory	4 (11.1%)	4 (11.1%)	1.000^F^
Gastrointestinal	8 (22.2%)	4 (11.1%)	0.343^F^
Sepsis	3 (8.3%)	5 (13.9%)	0.710^F^
Operative wound	-	2 (5.6%)	0.493^F^
Renal	3 (8.3%)	7 (19.4%)	0.307^F^
Death			0.151^F^
No	34 (94.4%)	29 (80.6%)	
Yes	2 (5.6%)	7 (19.4%)	

*=variables have missing; ^Q^=chi-square test of independence; ^F^=Fisher's exact test; ^L^=binary logistic model; ^W^=Wilcoxon Mann- Whitney test for independent samples; value of p>0,005; ICU= intensive care unit

Evaluating patients who died, only two variables were statistically significant between the two groups. The histological type of Lauren, where the diffuse pattern was predominant (100.0%) in group I, and the intestinal type (100.0%) in group II; and the tumor staging with IIIB (100%) tumors in group I and IB (40%) and IIB (60%) in group II (Table 4).

**TABLE 4:** Clinical, histopathological and postoperative complications of patients who died after radical gastrectomy for the treatment of gastric carcinoma (n=9)

Characteristics	Group I - < 65 years *(n=2)*	Group II - ≥ 65 years *(n=7)*	p-valor
Gender			0.444^F^
Female	-	4 (57.1%)	
Male	2 (100%)	3 (42.9%)	
Age (mean ± SD)	62.5 ± 0.7	80.4 ± 9.3	0.056^W^
ASA*			1.000^F^
II	1 (100%)	4 (66.7%)	
III	-	2 (33.3%)	
Smoking			1.000^F^
No	2 (100%)	5 (71.4%)	
Yes	-	2 (28.6%)	
Anesthetic technique*			1.000^F^
General	2 (100%)	4 (66.7%)	
General + block	-	2 (33.3%)	
Gastrectomy			1.000^F^
Total	1 (50%)	2 (28.6%)	
Subtotal	1 (50%)	5 (71.4%)	
Tumor location*			0.634^L^
Distal	-	2 (33.3%)	
Medial	1 (50%)	3 (50%)	
Proximal	1 (50%)	1 (16.7%)	
Laurén Classification*			<0.001^L^
Diffuse	1 (100%)	-	
Intestinal	-	5 (71.4%)	
Mixed	-	2 (28.6%)	
Final tumor staging*			<0.001^L^
IB	-	2 (40%)	
IIB	-	3 (60%)	
IIIB	1 (100%)	-	
Positive lymph node			1.000^F^
No	1 (50%)	5 (71.4%)	
Yes	1 (50%)	2 (28.6%)	
Postoperative complications			
Cardiorespiratory	1 (50%)	2 (28.6%)	1.000^F^
Gastrointestinal	1 (50%)	4 (57.1%)	1.000^F^
Sepsis	2 (100%)	5 (71.4%)	1.000^F^
Operative wound	-	-	-
Renal	1 (50%)	5 (71.4%)	1.000^F^

*=variables have missing; **=allows multiple responses; ^F=^Fisher exact test; ^L^=binary logistic model; ^W^=Wilcoxon Mann-Whitney test for independent samples; Value of p>0,005

The features of the nine patients that evolved to death are described in detail in Table 5. 

**TABLE 5:** Clinical, histopathological, and postoperative individual characteristics of patients who died after radical gastrectomy for the treatment of gastric carcinoma (n=9)

Patient	Age	Staging	ASA	Cause of Death	Gastrectomy type
1	88	IB	3	Sepsis	Subtotal
2	70	IIB	2	Sepsis	Subtotal
3	79	IIIB	2	Acute kidney failure	Subtotal
4	80	IIB	3	Sepsis	Subtotal
5	94	IIB	2	Unidentified shock	Subtotal
6	68	IB	2	Sepsis	Total
7	62	IIIB	2	Sepsis	Total
8	59	IIIB	2	Sepsis	Total
9	58	IIIB	1	Fistula	Total

ASA=American Society of Anesthesiologists.

## DISCUSSION

GC is a public health problem, accounting for many deaths, and age is considered one of the independent factors for the increase in the incidence of GC^25^ ^,5^. All patients were submitted to laparoscopy gastrectomy, regardless of stage. Some studies comparing open and laparoscopy gastrectomy for advanced gastric cancer (T2 or more) concluded that laparoscopy surgery is a feasible treatment strategy for advanced gastric tumors and that experienced surgeons can safely perform laparoscopy with D2 lymphadenectomy for advanced GC^3^. Our data indicated that, as in other countries, Brazilian surgeons perform more and more laparoscopy gastrectomy for advanced tumors^2^.

Although preoperative clinical evaluation is a common practice, it must consider specific clinical aspects in the elderly. Many abnormal laboratory findings are less valuable than history and physical examination in predicting postoperative morbidity^8^. Nelen et al.^17^, in a study with patients with GC, showed that one of the most striking features that distinguish young and old patients is the number of comorbidities that each presents. In their study, 72% of the male patients over 80 years old had comorbidities. In the study presented here, the number and the type of concomitant medications were used as an indirect measure to estimate comorbidities. The group of elderly had a higher number of concomitant medications. Data from the literature indicate that in the preoperative evaluation of patients with GC, age should not be the main criterion in which treatment decisions are made, but rather the presence of comorbidities^13^.

Corroborating this data, we analyzed the ASA classification. In this study, ASA 1, a healthy patient, was more common in group I patients. However, the ASA 2 means patients with severe systemic disease with functional limitation were more prevalent in group II^7^. The higher prevalence of ASA 2 can be justified by the high age group since, overall, chronic diseases begin in the elderly phase of life^24^. A study by Tegels et al.^23^ discussed the ASA classification about gastrectomy in the elderly, reporting the classification's non-specificity and ephemerality in this age group. Other studies have shown similar rates of postoperative morbidity and mortality among young and elderly adults, not directly related to the ASA classification, but the stage of cancer^6^. Other authors, otherwise, have reported that the ASA 2 score indicating is an established predictor of adverse post-surgery outcomes in patients of all ages, but it does not specify age as a risk factor^27^. 

GC in young and old patients presents different clinical, histological, and molecular characteristics. In the present study, group I presented predominance of the diffuse type according to Lauren's histological classification and group II the intestinal type. Similar results from a South Korea study showing a higher prevalence of diffuse-type in younger and intestinal-type patients in older patients^22^. 

The extent of resection in GC depends on the tumor's size and location, the depth of its invasion, and the histological type. In general, total gastrectomy is performed in proximal tumors, and the subtotal in distal ones, associated with D2 lymphadenectomy^11^. In both groups, subtotal gastrectomy was the mandatory surgery for most patients, probably due to the higher prevalence of distal tumors. 

In both groups, free margins were obtained in most cases. A meta-analysis assessed whether total gastrectomy would provide better outcomes than subtotal gastrectomy for distal gastric cancer and showed that, despite postoperative mortality being similar, total gastrectomy for distal gastric cancer harmed overall survival at five years. Therefore, subtotal gastrectomy remains a recommendation for distal gastric cancer, either because of the absence of randomized multicenter trials or the limited size of studies that follow long-term surgical outcomes^20^.

In a meta-analysis, Pan et al.19, including 3275 patients with GC, observed that in the geriatric group, the number of resected lymph nodes was lower than that of young adults but without a change in the rate of overall survival expectancy. This shows that lower oncological radicality in this population and the non-change in overall survival may be related to the absence of comorbidities. More radical surgical procedures with resection of a high number of lymph nodes were associated with higher postoperative complications.

It was observed in the present study that, in both groups, the extent of lymphadenectomy was similar. According to the American Joint Committee on Cancer (AJCC) guidelines, the minimum number of lymph nodes for adequate tumor staging was reached, also indicating the oncological radicality of the surgeries^9^. This data shows that the elderly population may have the same chances of cure and can be submitted to procedures similar to younger patients.

Previous studies have identified age as a predictive factor of postoperative morbidity after gastrectomy^14^. On the other hand, some authors did not observe this relationship and reported morbidity and mortality rates in elderly patients, similar to young patients^18^. Kim et al.^13^ evaluated the surgical procedure in the elderly, reaffirming the safety of subtotal gastrectomy in this age group. Postoperative complications were observed but like those expected for younger patients^13^. This evidence was also observed in the present study since there was no statistically significant difference between days of ICU stay, hospitalization time, number of complications, and death among the assessed groups.

Concerning the patients that died, there was no relative difference between the analyzed variables (gender, ASA, smoking history, anesthetic technique, tumor location, and positive lymph node presence) between the two groups, except the histological type of Laurén and the final staging. More common distal tumors in the patients evolved to death in group I and medial and proximal in group II. Tumor staging IIIB was the most common in group I and group II tumor stages IB and IIB. Regardless of age, tumor staging is an independent prognostic factor for overall survival and cancer-specific survival^16^, but in this study, long-term survival was not evaluated but instead in the postoperative period. Even statistically not different, the postoperative death rate of 19.4% in the elderly group is very expressive. The Elderly is regarded as being at increased risk during major abdominal surgery because of a lack of functional reserve and an increased number of comorbidities^21^.

When postoperative complications were evaluated, all patients in group I had sepsis. A Chinese meta-analysis evaluated 2482 patients undergoing GC gastrectomy, and postoperative complications occurred in 8.9% of the cohort, with the most common being those related to infectious processes such as intra-abdominal infection, anastomotic extravasation, ascites, intra-abdominal bleeding, infection pulmonary and pleural effusion^30^.

Group II presented sepsis associated with renal complications. Acute kidney injury is known to be a complication associated with high morbidity and mortality in hospitalized patients^28^. It is not only one of the most common postoperative complications, especially after gastrectomy, but it is also associated with in-hospital mortality, long-term mortality after surgery, and an increased risk of progression to chronic kidney disease and renal failure^12^.

Some limitations are inherent to this study, mainly because it is a retrospective design with a small sample. Pre-anesthetic evaluations were performed in an out-of-hospital setting leading to data loss.

## CONCLUSIONS

The results of this study indicate that laparoscopic gastrectomy, performed by qualified and well-trained surgeons, is a safe procedure, with no differences in morbidity and length of hospitalization among young and elderly patients. Advanced tumor staging and comorbidities were related to surgical mortality. The radicality of surgical treatment was equal regardless of age.
